# Effect of a Combination of Prebiotic Supplements Based on Fucus and Kelp on the Gut Microbiome of Mice with Induced Inflammation

**DOI:** 10.3390/microorganisms14030592

**Published:** 2026-03-06

**Authors:** Anatoly A. Khitrov, Inna Yu. Burakova, Yuliya D. Smirnova, Svetlana V. Pogorelova, Egor A. Chirkin, Polina D. Morozova, Daniil A. Garmonov, Elena V. Ozhimkova, Mikhail Yu. Syromyatnikov, Olga S. Korneeva

**Affiliations:** 1 Department of Biochemistry and Biotechnology, Voronezh State University of Engineering Technologies, 19 Revolutsii Prospect, Voronezh 394036, Russia; anatoly.khitrov@gmail.com (A.A.K.); vitkalovai@inbox.ru (I.Y.B.); dyd16@mail.ru (Y.D.S.); zubkowa.sweta@gmail.com (S.V.P.); chirkin@bio.vsu.ru (E.A.C.); ms.cloud00.00@mail.ru (P.D.M.); korneeva-olgas@yandex.ru (O.S.K.); 2 Department of Genetics, Cytology and Bioengineering, Voronezh State University, 1 Universitetskaya Sq., Voronezh 394018, Russia; 3 Department of Biotechnology, Chemistry and Standardization, Tver State Technical University, Nikitina Street, 22, Tver 170026, Russia; garmonov.daniil@icloud.com (D.A.G.); eozhimkova@mail.ru (E.V.O.)

**Keywords:** prebiotics, *Laminaria digitata*, *Fucus vesiculosus*, inflammation, lipopolysaccharide, sequencing, DNBSEQ-G50 platform

## Abstract

Gut microbiota imbalances can lead to the development of various inflammatory diseases in the body. The development of drugs aimed at maintaining intestinal health is a key area of biotechnology. Algae-based prebiotics are one such drug. The aim of this study was to conduct a comparative analysis of the fecal microbiota of *Mus musculus* with and without a prebiotic supplement. We studied the effects of enzymatically processed *Laminaria digitata* and *Fucus vesiculosus* seaweeds on the gut microbiome of mice with induced inflammation using DNBSEQ-G50 sequencing. The results showed that these prebiotic supplements can reduce the impact of inflammation on the intestine. An increase in the relative abundance of *Anaerostipes rhamnosivorans*, *Dysosmobacter welbionis*, *Akkermansia muciniphila*, *Flavonifractor plautii*, and a decrease in *Longicatena caecimuris* relative to the LPS group were observed. Furthermore, enzymatically processed algae were found to increase the relative abundance of gut bacterial metabolic pathways responsible for glucose breakdown. Thus, both enzymatically processed and unprocessed algae-based prebiotic supplements restored gut microbiome composition and gut morphology in LPS-exposed mice, as confirmed by microbiome analysis and histological examination.

## 1. Introduction

It is known that disruption of intestinal microbiota homeostasis can lead to the development of intestinal disorders such as inflammatory diseases, immunodeficiency, hypertension, type 2 diabetes, obesity, and cancer [1]. Algae are a promising resource for renewable nutrition, which increases the need for the development of dietary supplements based on them [2]. It is known that adding microalgae-based supplements to animal diets can improve both intestinal physiology and host immunity, preventing intestinal damage in various inflammatory conditions [3]. Several species of wild microalgae are widely used in human food: *Arthrospira platensis*, *Chlorella vulgaris*, and *Aphanizomenon* [4]. Previously, it was shown that adding *Fucus vesiculosus* to the diet of mice improves the health of the intestinal microbiota, enriching it with bacteria capable of breaking down fiber and producing short-chain fatty acids [5]. Furthermore, fucoidans isolated from *Fucus vesiculosus* have been shown to exert an anticancer effect in a rat model of colorectal cancer [6]. Brown algae, such as *Fucus* and *Laminaria*, are used in the treatment of metabolic diseases and intestinal barrier damage [7]. Furthermore, studies examining the effects of *Laminaria* powder and fucoidan on the intestinal microbiota have shown that both substances have a positive effect on both the structure of the intestinal microbiota and the habitat of large yellow croakers [8].

Enzymatic hydrolysis is a preferred method for separating bioactive peptides, capable of improving the key properties of peptides: activity and bioavailability [9]. Thus, there is great potential in the research and development of enzymatically hydrolyzed microalgae as sources of high-quality and stable protein for human food products and dietary supplements [10]. However, studies on the prebiotic activity of *Fucus vesiculosus* and *Laminaria digitata* in modulating the composition of the gut microbiome under conditions of lipopolysaccharide-induced inflammation are limited and do not provide comprehensive data. Thus, the main aim of our study was to identify the effects of enzymatically treated algae and fucus on the gut microbiome.

## 2. Materials and Methods

### 2.1. Objects of Research

The studies were conducted on male *Mus musculus* of the C57BL/6 line obtained from the Stolbovay nursery (Moscow region, Russia).

The studied mice were kept in accordance with “Guidelines for the maintenance and care of laboratory animals. Rules for equipping premises and organizing procedures.”

A total of 36 male mice were used. Laboratory mice were then divided into 4 groups: a control group (*n* = 10); a control group injected with lipopolysaccharide (LPS) (*n* = 10); a group that received a prebiotic based on enzymatically processed *Fucus vesiculosus* and *Laminaria digitata* in the form of a gel (prebiotic supplement 1) and which was administered LPS (*n* = 8); a group supplemented with a prebiotic based on non-hydrolyzed *Fucus vesiculosus* and *Laminaria digitata* in the form of a powder (Prebiotic supplement 2) and injected with LPS (*n* = 8).

The experimental protocol included a three-week period of prebiotic administration and a one-week course of LPS injections. Samples of brown seaweed were collected in the White Sea. Thalli of the brown seaweed *Laminaria digitata* (order Laminariales) were collected in the coastal zone of Maly Zhuzhmuy Island. Thalli of the brown seaweed *Fucus vesiculosus* (order Fucales) were collected in the coastal zone of Maly Zhuzhmuy Island. After collection, the thalli were placed in an isothermal thermal box for delivery to the laboratory. During transportation, the temperature was maintained at 4 ± 2 °C. Samples of brown seaweed in the laboratory were stored in a sealed package at a temperature of 4 ± 2 °C.

Prebiotic supplement 1 preparation: Before enzymatic hydrolysis, the thalli of the brown seaweeds under study were washed several times with deionized water to remove sea salt and contaminants. After washing, the thalli were air-dried and freeze-dried in a FreeZone 2.5 L lyophilizer (Labconco, Kansas, MO, USA) to constant humidity. Freeze-drying mode: −52 °C, pressure 50 Pa, duration 24 h. The dried samples were ground in a laboratory mill for 60 s. A fraction with a particle size of 50–100 μm was selected for the experiments. To perform enzymatic hydrolysis, 0.1 M citrate buffer characterized by a pH value of 5.0 pH units was selected. The following enzyme preparations were selected for enzymatic hydrolysis: complex enzyme preparation “Ceremix Plus MG” (Novozymes, Bagsværd, Denmark) and enzyme preparation “Celluclast 1.5 L” (Novozymes, Bagsværd, Denmark). Complex enzyme preparation “Ceremix Plus MG” (Novozymes, Denmark) is characterized by endo-1,4-xylanase, endo-1,3(4)-β-glucanase, neutral protease and α-amylase activities. The enzyme preparation “Celluclast 1.5 L” (Novozymes, Bagsværd, Denmark) is characterized by the presence of cellulase activity. Micronized thallus of the brown seaweed *Laminaria digitata* and micronized thallus of the brown seaweed *Fucus vesiculosus* were suspended in 300 cm^3^ of 0.1 M citrate buffer (5.0 pH units) until the ratio of dry weight of micronized thalli of brown seaweed: 0.1 M citrate buffer was 1:9. In this case, the citrate buffer was preheated to 50 °C. The mass ratio of micronized thalli of the brown seaweed *Laminaria digitata* and micronized thalli of the brown seaweed *Fucus vesiculosus* in the system was 50:50 (d.w.). Suspension was carried out with constant stirring by an overhead stirrer with an anchor stirrer with a rotation speed of 500 rpm. The end of suspension was considered to be the obtaining of a homogeneous viscous mass of brown color, which took on average 30 min. Upon completion of suspension, enzyme preparations were added at the following dosages: “Ceremix Plus MG” 5% of the dry matter of micronized thalli of brown seaweed in the system, “Celluclast 1.5 L” (Novozymes, Bagsværd, Denmark) 5% of the dry matter of micronized thalli of brown seaweed in the system. In this case, “Ceremix Plus MG” was pre-dissolved in 10 cm^3^ of 0.1 M citrate buffer (5.0 pH units) at 50 °C. The liquid enzyme preparation “Celluclast 1.5 L” was added without pre-dissolution in the buffer solution and without heating. Hydrolysis was carried out for 24 h with constant stirring by an overhead stirrer with an anchor stirrer with a rotation speed of 500 rpm and maintaining the temperature at 50 °C. To prevent evaporation, a laboratory beaker was equipped with a lid with a rotation unit for connecting the overhead stirrer. Enzymatic hydrolysis was stopped by heating the system to 90 °C and then maintaining it at this temperature for 10 min. Then the hydrolysate was transferred to an ice bath for cooling. The resulting hydrolysate was used for further studies.

Prebiotic supplement 2 preparation: the thalli of the brown seaweed under study were washed several times with deionized water to remove sea salt and contaminants. After washing, the thalli were air-dried and freeze-dried in a FreeZone 2.5 L freeze dryer (Labconco, Kansas, MO, USA) to constant humidity. Freeze-drying mode: −52 °C, pressure 50 Pa, duration 24 h. The dried samples were ground in a Stegler LM-1000 laboratory mill (STEGLER, Kunshan, China) for 60 s. A fraction with a particle size of 50–100 μm was selected for the experiments. The ratio of micronized thalli of *Laminaria digitata*: *Fucus vesiculosus* in Prebiotic supplement 2 was 50:50, the full chemical composition is presented in the table below (Table 1).

Prebiotic supplement dosages were selected based on preliminary research to ensure ease of administration and to reflect typical practical use of the liquid and powder forms.

The drug “Pyrogenal” 100 μg/mL was used as a lipopolysaccharide (LPS) for the study. For injections, LPS was mixed with physiological solution in a ratio of 1:1 and administered intraperitoneally at 200 μL daily during the last week of the experiment.

Prebiotics were pre-mixed with distilled water before adding to the feed in a ratio of 400 μL of prebiotic per 1.75 mL of water per mouse per day (Prebiotic supplement 1) and 25 mg of prebiotic per 1.75 mL of water per mouse per day (Prebiotic supplement 2). All groups of mice received a portion of feed at a rate of 5 g/mouse [11]. Prebiotic supplement 1 (enzymatically hydrolyzed gel, 10% solids) provided 40 mg dry matter per mouse per day (1794 mg/kg/day). Prebiotic supplement 2 (non-hydrolyzed powder) was administered at 25 mg per mouse per day (1121 mg/kg/day).

The experiment lasted for 3 weeks, at the end of each week, as well as before the start of the experiment, a physiological test “open field” was carried out.

### 2.2. Histological Study

Histological analysis was performed according to the protocol from our previous work [12].

### 2.3. Generation of Sequencing Libraries on the DNBSEQ-G50 Platform

The study was conducted using metagenomic shotgun sequencing on the DNBSEQ-G50 platform [13].

At the first stage, total DNA was isolated from each obtained stool sample using a commercial MetaGen kit (Synthol, Moscow, Russia) in accordance with the manufacturer’s protocol. The concentration and purity of each sample were measured using a Nano-500 fluorimeter (Hangzhou Allsheng Instruments Co., Ltd., Hangzhou, China) and a commercial Equalbit 1x dsDNA HS kit (Vazyme, Nanjing, China).

After that, fragmentation was performed, with subsequent ligation of barcodes, all these operations were carried out using the kits: MGIEasy Fast FS Library Prep Module (MGI, Shenzhen, China) and MGIEasy UDB Adapter Kit (MGI, Shenzhen, China), based on the protocol declared by the manufacturer. Upon completion of this stage, the quality of the obtained PCR products was assessed based on the electrophoresis method in 2% agarose gel. The concentration of the products was also assessed using a Nano-500 fluorimeter (Hangzhou Allsheng Instruments Co., Ltd., Hangzhou, China) and an Equalbit 1x dsDNA HS Assay Kit (Vazyme, Nanjing, China). Based on the results obtained, PCR products were pooled, with subsequent measurement of pool concentrations. At the next stage, the pools were circularized to obtain single-stranded DNA, the operations were carried out based on the protocol of the manufacturer of the commercial kit MGIEasy Dual Barcode Circularization Module (MGI, Shenzhen, China). Subsequently, quality control was also performed by measuring the concentration using a Nano-500 fluorimeter (Hangzhou Allsheng Instruments Co., Ltd., Hangzhou, China) and a QuDye ssDNA Assay Kit (Lumiprobe, Moscow, Russia). Based on the quality control results, the volume of each pool was calculated for combining into a superpool. The concentration of the superpool was also controlled and the volume of the superpool was calculated to be 60 fmol.

To start the DNBSEQ-G50 sequencer (MGI, Shenzhen, China), DNB were formed using the commercial DNBSEQ-G50RS High-throughput Sequencing Kit FCL PE100/FCS PE150 (MGI, Shenzhen, China). Then, the concentration of the obtained DNB was measured using a Nano-500 fluorimeter (Hangzhou Allsheng Instruments Co., Ltd., Hangzhou, China) and a commercial QuDye ssDNA Assay Kit (Lumiprobe, Moscow, Russia). After that, DNB were placed in the sequencer using a DNBSEQ-G50RS Sequencing Flow Cell FCL (MGI, Shenzhen, China), as well as a cartridge PE100 with reagents from the DNBSEQ-G50RS High-throughput Sequencing Kit FCL PE100/FCS PE150 (MGI, Shenzhen, China) kit. All operations were carried out based on the manufacturer’s protocols.

### 2.4. Bioinformatic and Statistical Analysis

The quality of the raw reads was assessed using FastQC [14]. Subsequent filtering included the removal of technical sequences and low-quality nucleotides (Q < 30) using the fastp tool [15]. To exclude contamination with human and mouse genomes, the reads were mapped to the corresponding reference genomes using Bowtie2 [16]. The taxonomic composition of bacteria, viruses, and eukaryotes in the microbiomes of the samples was determined using MetaPhlAn4 [17]. Functional profiling of metabolic pathways was performed using HUMAnN3.0 [18].

Statistical data processing was carried out in the R environment, the RStudio program (2025.05.0). Alpha diversity was estimated using the Shannon index, and the Bray–Curtis dissimilarity metric was used to analyze beta diversity. Differences in alpha diversity were assessed using the nonparametric Mann–Whitney test. The ADONIS (999 permutations) function was used to assess differences in diversity between groups. To ensure that observed effects reflected genuine location shifts rather than unequal within-group dispersion, we complemented ADONIS with PERMDISP (999 permutations). Differential abundance analysis of species was performed using the MaAsLin2 package [19], which used a multivariate regression model. Data on metabolic pathways were calculated using the Kruskal–Wallis test and Bonferroni correction. Observed power for key outcomes was estimated post hoc with the pwr package via Cohen’s d. An adjusted *p*-value ≤ 0.05 was considered statistically significant. Results are presented as mean ± SD.

## 3. Results

### 3.1. Histology

**Figure 1:**
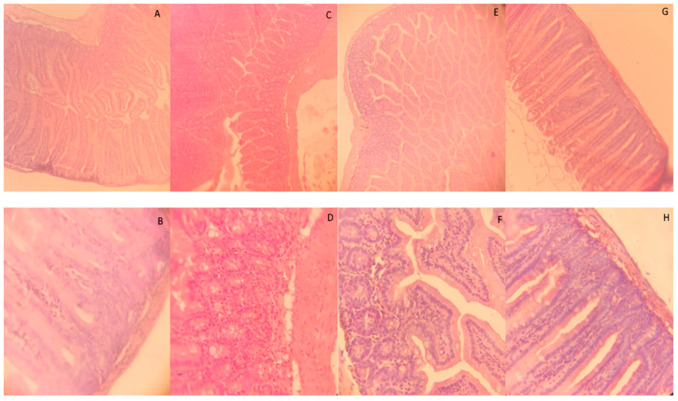
Morphological structure of the mouse intestine. Magnification: (**A**,**C**,**E**,**G**) 100×, (**B**,**D**,**F**,**H**) 400×. Hematoxylin and eosin staining: (**A**,**B**) “Control” group; (**C**,**D**) “LPS” group; (**E**,**F**) “Prebiotic supplement 1” group; (**G**,**H**) “Prebiotic supplement 2” group.

Histological studies showed that the control group intestine had a generally accepted structure: finger-shaped villi with a brush border, goblet cells were located singly among the border cells, crypts were formed and lay in the proper layer of the mucous membrane, the proper layer of the mucous membrane was built of loose fibrous unformed connective tissue, the muscular membrane was represented by the outer and inner muscular layers. In the “LPS” group, multiple destructive changes in the intestinal mucosa were noted, expressed in the violation of the structural integrity of the villi, the presence of cellular infiltration and areas of connective tissue growth, which indicates the occurrence of an inflammatory process in the intestine. In the “Prebiotic supplement 1” group, the overall picture of the morphological structure of the intestine was unchanged, but it is worth noting the presence of a small number of areas with cellular infiltration. In the “Prebiotic supplement 2” group, the histological structure of the intestine was most similar to the control group, but the intestinal mucosa was characterized by the highest villi with a large number of goblet cells, which improves the functional state of the intestine. Figure 1 shows the morphological structure of the intestine of mice in the study groups.

Histological studies also showed that the “Prebiotic supplement 2” group demonstrated the highest values of villus height (370 μm on average) and crypt depth (40 μm on average), which may indicate a stimulating effect of this combination on the morphological structure of the intestine. The LPS group had the smallest villus width, which indicates a violation of the structural organization of the intestinal mucosa. The control group demonstrated average values. Correlation analysis revealed a moderate positive correlation (r ≈ 0.6) between the height and depth of the crypts, which may indicate the interrelated development of these structures, as well as a weak correlation between the width and height of the villi (r ≈ 0.3), which indicates the independence of these parameters in some groups. The villus height values were statistically significant between the “Prebiotic supplement 2” group and the comparison groups at *p* ≤ 0.05; The villus width values are statistically significant in the LPS group compared to other groups at *p* ≤ 0.05; the crypt depth shows significantly higher values in the Prebiotic supplement 2 group at *p* ≤ 0.05 compared to other groups (Table 2).

### 3.2. Sequencing Results on the DNBSEQ-G50 Platform

**Figure 2:**
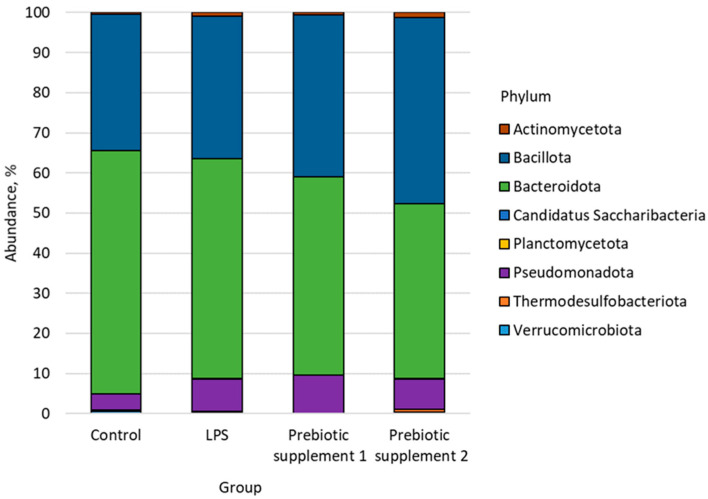
Bacterial types found in the faecal microbiome of the study groups.

Analysis of the fecal microbiome of laboratory mice from the Control, LPS, Prebiotic supplement 1 and Prebiotic supplement 2 groups allowed us to identify 8 phyla, 17 classes, 25 orders, 34 families, 60 genera and 115 species of bacteria (Figure 2). 

**Figure 3:**
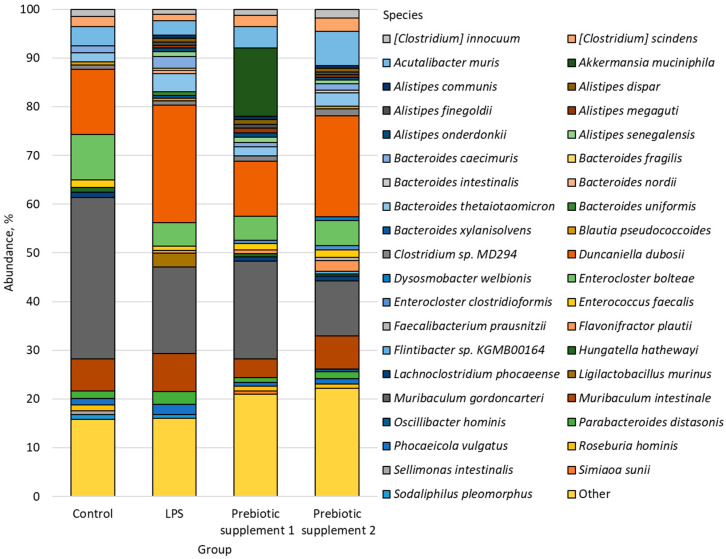
Bacterial species found in the microbiome of the study groups.

We conducted a comparative analysis of the intestinal microbiome of the study groups, relative to Control, and identified 39 most common species, the number of which exceeded 0.5%, all other species were grouped as “Others” (Figure 3).

**Figure 4:**
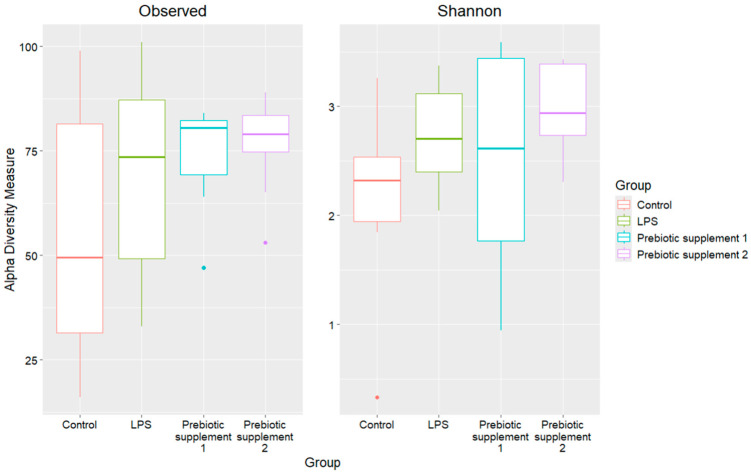
Alpha diversity of the microbiome of the study groups.

An alpha diversity analysis was also performed using the observed species diversity measures and the Shannon index (Figure 4, Table 3). No statistically significant differences were found.

**Figure 5:**
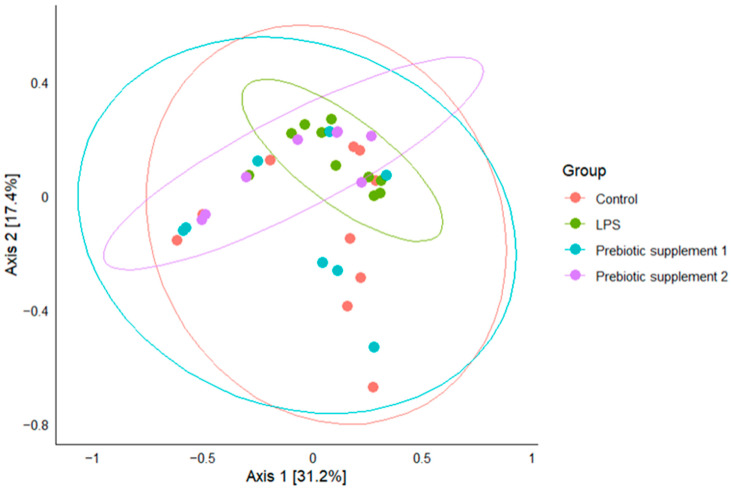
Principal coordinates analysis (PCoA) plot of beta diversity based on the Bray–Curtis scale between study groups.

A global beta diversity analysis (999 permutations) revealed a nonsignificant effect of group on microbial community structure (R^2^ = 0.137, F = 1.697, *p* = 0.071). Pairwise comparisons revealed a statistically significant difference between the LPS and Prebiotic supplement 1 groups (*p* = 0.044, R^2^ = 0.128, F.Model = 2.351). The homogeneity of multivariate variances differed significantly between groups (*p* = 0.017), suggesting that differences between groups reflect both changes in composition and variations in community variance (Figure 5). The statistical power calculation showed 59%, but Cohen’s d effect itself shows that the results are significant (d = 0.77).

**Figure 6:**
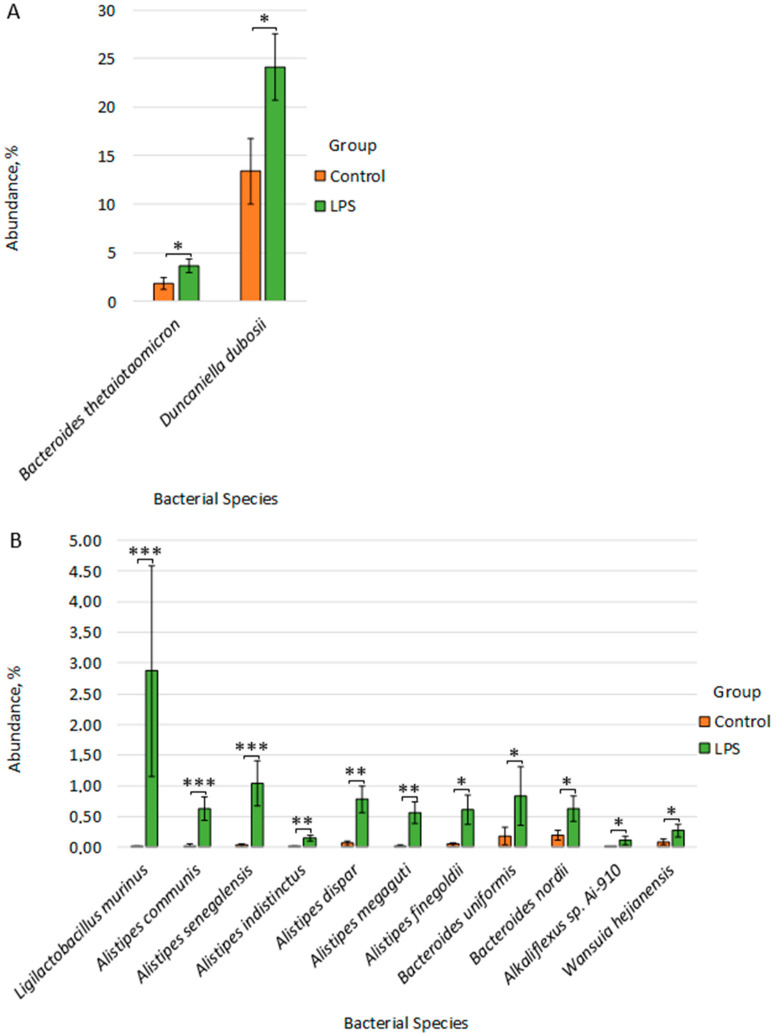
(**A**,**B**) Differences in fecal microbiome composition between Control and LPS groups. * *p* ≤ 0.05, ** *p* ≤ 0.01, *** *p* ≤ 0.001.

Differential abundance analysis revealed statistically significant differences at the species level between the Control group and the LPS group (Figure 6A,B). Thus, in the LPS group, compared to the Control group, we observed an increase in the number of species: *Ligilactobacillus murinus* (2.874% ± 1.715 vs. 0.010% ± 0.008, *p* = 6.22 × 10^−5^), *Alistipes communis* (0.629% ± 0.186 vs. 0.022% ± 0.022, *p* = 6.12 × 10^−4^), *Alistipes senegalensis* (1.038% ± 0.369 vs. 0.031% ± 0.023, *p* = 9.15 × 10^−4^), *Alistipes indistinctus* (0.151% ± 0.044 vs. 0.008% ± 0.008, *p* = 0.001), *Alistipes dispar* (0.781% ± 0.219 vs. 0.066% ± 0.038, *p* = 0.006), *Alistipes megaguti* (0.568% ± 0.173 vs. 0.017% ± 0.012, *p* = 0.006), *Bacteroides thetaiotaomicron* (3.662% ± 0.730 vs. 1.857% ± 0.569, *p* = 0.014), *Alistipes finegoldii* (0.615% ± 0.238 vs. 0.051% ± 0.024, *p* = 0.012), *Duncaniella dubosii* (24.117% ± 3.461 vs. 13.407% ± 3.399, *p* = 0.030), *Bacteroides uniformis* (0.836% ± 0.474 vs. 0.181% ± 0.143, *p* = 0.036), *Bacteroides nordii* (0.623% ± 0.209 vs. 0.197% ± 0.079, *p* = 0.029), *Alkaliflexus* sp. *Ai-910* (0.111% ± 0.073 vs. 0.004% ± 0.003, *p* = 0.033), *Wansuia hejianensis* (0.268% ± 0.100 vs. 0.085% ± 0.052, *p* = 0.041). All detected differences showed statistical power >97%, which confirms the high reliability of the detected differences, despite the relatively small sample size.

Statistically significant differences were also found at the species level between the LPS group and the Prebiotic supplement 1 group. Thus, in the Prebiotic supplement 1 group, compared to the LPS group, we observed an increase in the number of species: *Akkermansia muciniphila* (14.033% ± 9.051 vs. 0.031% ± 0.015, *p* = 0.004), *Butyricimonas virosa* (0.170% ± 0.054 vs. 0.034% ± 0.012, *p* = 0.015), *Blautia* sp. *NBRC 113351* (0.155% ± 0.076 vs. 0.026% ± 0.014, *p* = 0.034), *Coprococcus* sp. *ART55/1* (0.116% ± 0.051 vs. 0.027% ± 0.023, *p* = 0.040), *Longicatena caecimuris* (0.265% ± 0.128 vs. 0.051% ± 0.029, *p* = 0.041), *Anaerostipes rhamnosivorans* (0.143% ± 0.077 vs. 0.019% ± 0.017, *p* = 0.048).

**Figure 7:**
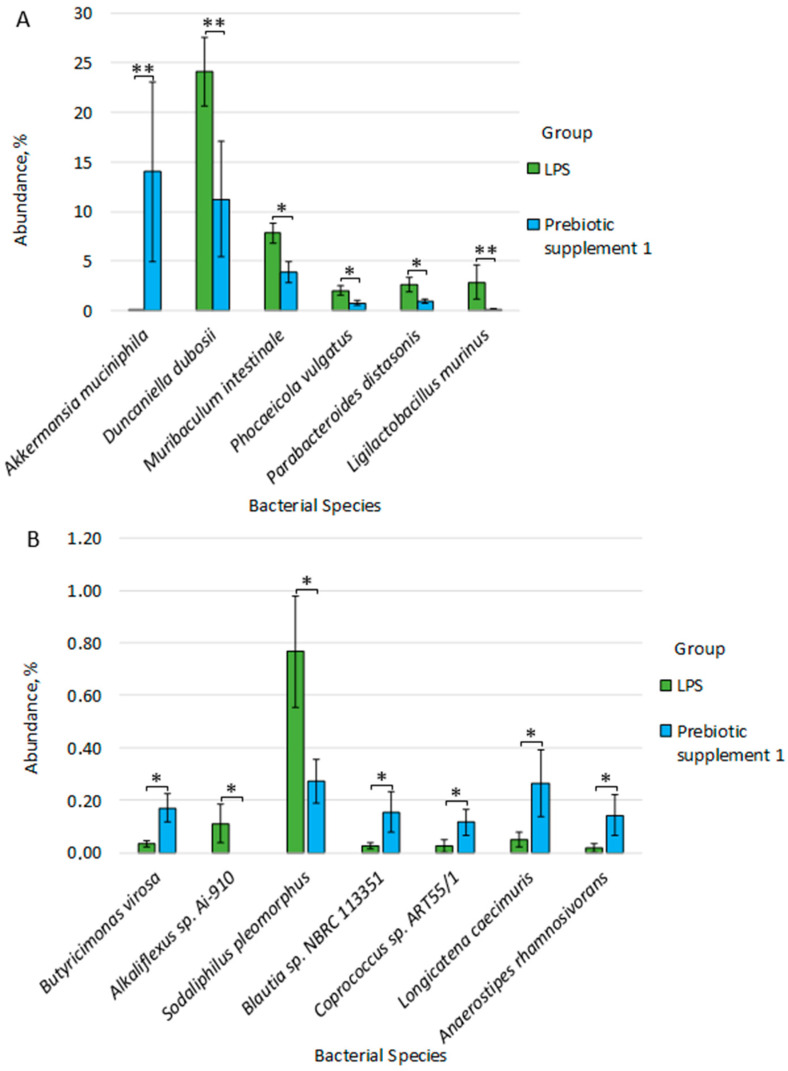
(**A**,**B**) Differences in fecal microbiome composition between LPS and Prebiotic supplement 1 groups. * *p* ≤ 0.05, ** *p* ≤ 0.01.

In contrast, the abundance of the following species was significantly higher in the LPS group compared to the Prebiotic supplement 1 group: *Duncaniella dubosii* (24.117% ± 3.461 vs. 11.232% ± 5.808, *p* = 0.009), *Ligilactobacillus murinus* (2.874% ± 1.715 vs. 0.133% ± 0.063, *p* = 0.008), *Alkaliflexus* sp. *Ai-910* (0.111% ± 0.073 vs. 0, *p* = 0.016), *Muribaculum intestinale* (7.833% ± 0.984 vs. 3.909% ± 1.052, *p* = 0.029), *Sodaliphilus pleomorphus* (0.767% ± 0.211 vs. 0.273% ± 0.082, *p* = 0.042), *Phocaeicola vulgatus* (2.023% ± 0.476 vs. 0.765% ± 0.259, *p* = 0.041), *Parabacteroides distasonis* (2.660% ± 0.728 vs. 0.990% ± 0.219, *p* = 0.035) (Figure 7A,B). The statistical power to estimate differences at the species level was greater than 90%, which also indicates the high significance of the results even with a small sample.

**Figure 8:**
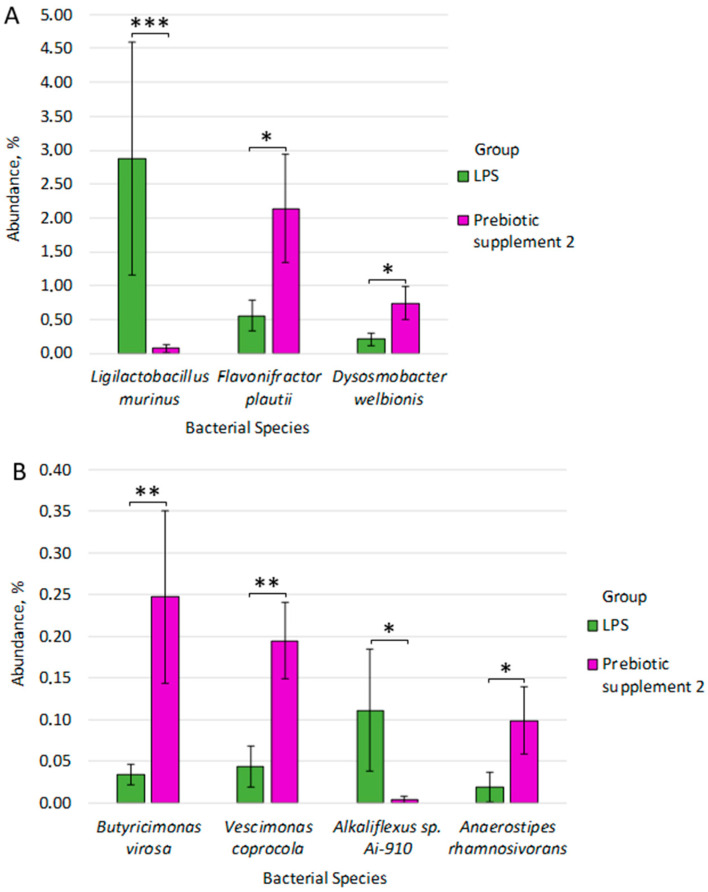
(**A**,**B**) Differences in fecal microbiome composition between LPS and Prebiotic supplement 2 groups. * *p* ≤ 0.05, ** *p* ≤ 0.01, *** *p* ≤ 0.001.

A statistically significant predominance of species was observed in the Prebiotic supplement 2 group compared to the LPS group: *Butyricimonas virosa* (0.247% ± 0.103 vs. 0.034% ± 0.012, *p* = 0.009), *Vescimonas coprocola* (0.195% ± 0.046 vs. 0.043% ± 0.024, *p* = 0.009), *Flavonifractor plautii* (2.140% ± 0.804 vs. 0.554% ± 0.224, *p* = 0.014), *Anaerostipes rhamnosivorans* (0.099% ± 0.041 vs. 0.019% ± 0.017, *p* = 0.042), *Dysosmobacter welbionis* (0.739% ± 0.243 vs. 0.210% ± 0.091, *p* = 0.033) (Figure 8A,B).

In contrast, the LPS group had a significantly greater abundance of *Ligilactobacillus murinus* compared to the Prebiotic supplement 2 group: *Ligilactobacillus murinus* (2.874% ± 1.715 vs. 0.069% ± 0.064, *p* = 4.60 × 10^−4^), *Alkaliflexus* sp. *Ai-910* (0.111% ± 0.017 vs. 0.099% ± 0.041, *p* = 0.043) (Figure 8A,B). Taxa with the strongest effects (e.g., *Ligilactobacillus murinus*, d = 3.9) showed a power of 71–98%, while taxa close to the significance threshold (e.g., *Alkaliflexus* sp. *Ai-910*, d = 2.11; *Anaerostipes rhamnosivorans* d = 2.12; *Dysosmobacter welbionis*, d = 2.24) had a power of ~60%, despite high Cohen’s d values.

**Figure 9:**
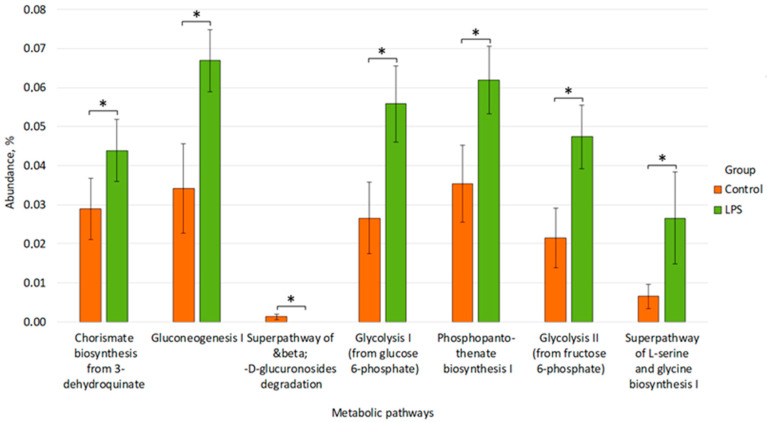
Differences in the prevalence of metabolic pathways between the Control and LPS groups. * *p* ≤ 0.05.

After analyzing the data on metabolic pathways, the following statistically significant results were revealed. In the LPS group, compared to Control, there was an increase in the activity of such metabolic pathways as GLUCONEO-PWY: gluconeogenesis I (0.044 ± 0.008 vs. 0.029 ± 0.008, *p* = 0.018), GLUCUROCAT-PWY: superpathway of &beta;-D-glucuronosides degradation (0.067 ± 0.008 vs. 0.034 ± 0.011, *p* = 0.024), PANTO-PWY: phosphopantothenate biosynthesis I (from glutamate) pathway (0.056 ± 0.010 vs. 0.027 ± 0.009, *p* = 0.028), PWY-5484: glycolysis II (from fructose 6-phosphate) (0.062 ± 0.009 vs. 0.035 ± 0.010, *p* = 0.032), PWY-6163: chorismate biosynthesis from 3-dehydroquinate (0.047 ± 0.008 vs. 0.021 ± 0.008, *p* = 0.028), SER-GLYSYN-PWY: superpathway of L-serine and glycine biosynthesis I (0.027 ± 0.012 vs. 0.007 ± 0.003, *p* = 0.049). However, inhibition of the GLYCOLYSIS: glycolysis I (from glucose 6-phosphate) pathway was observed after exposure to LPS injections (1.29 × 10^−3^ ± 7.73 × 10^−4^ vs. 0, *p* = 0.032) (Figure 9).

**Figure 10:**
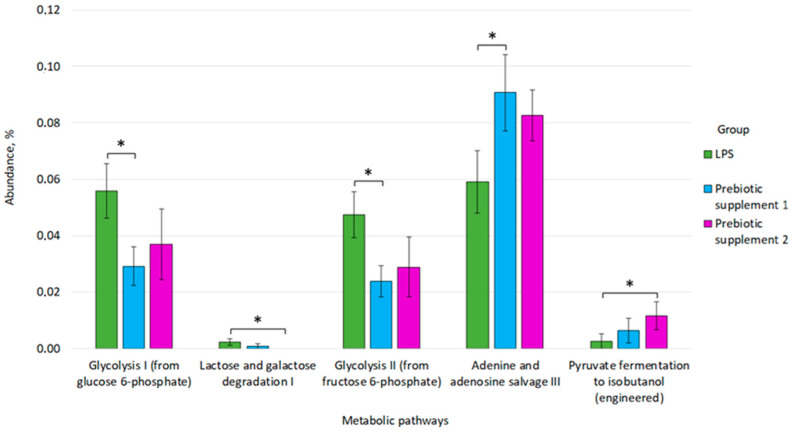
Differences in the prevalence of metabolic pathways between the LPS, Prebiotic supplement 1 and Prebiotic supplement 2 groups. * *p* ≤ 0.05.

Also after taking prebiotic supplement 1, a decrease in the activity of the following metabolic pathways was recorded, relative to the LPS group: Glycolysis I (from glucose 6-phosphate) (0.059 ± 0.010 vs. 0.029 ± 0.007, *p* = 0.036), Glycolysis II (from fructose 6-phosphate) (0.047 ± 0.008 vs. 0.024 ± 0.005, *p* = 0.043) and an increase in the activity of the Adenine and adenosine salvage III pathway (0.091 ± 0.014 vs. 0.059 ± 0.011, *p* = 0.049) (Figure 10). In turn, after taking prebiotic supplement 2, there was an increase in the activity of the Pyruvate fermentation to isobutanol (engineered) metabolic pathway (0.011 ± 0.005 versus 0.003 ± 0.003, *p* = 0.035) and a decrease in the Lactose and galactose degradation I pathway (0.002 ± 0.001 versus 0, *p* = 0.041) relative to the LPS group (Figure 10).

## 4. Discussion

Modern science is actively studying various chemical compounds that demonstrate prebiotic activity and potential therapeutic benefits [20,21]. In our study, we analyzed the prebiotic properties of the algae *Laminaria digitata* and *Fucus vesiculosus*.

Based on the results of the histological study, it can be assumed that the prebiotic based on *Fucus vesiculosus* and *Laminaria digitata* in powder form is able to have the most pronounced positive effect on the intestinal morphology, increasing the height of the villi and the depth of the crypts. While LPS negatively affects the morphology of the intestine, causing inflammatory and degenerative processes. In addition, it was found that the studied groups of mice: “Prebiotic supplement 1” and “Control” show intermediate results. Correlation analysis confirms the relationship between the height of the villi and the depth of the crypts, which is important for understanding the mechanism of adaptation processes in the intestine in response to various factors.

The analysis of the obtained results showed bacterial changes in the gut microbiome of mice. The bacterial species *Ligilactobacillus murinus*, isolated from the intestinal tract of mice, is known for its antibacterial properties against clinical and zoonotic pathogens, as well as bacteriolytic enzymes [22]. It is noted that the abundance of *L. murinus* in the host body positively correlates with improved metabolic parameters and a healthier gut microbiota, while a decrease in numbers may be associated with aging, as well as metabolic disorders [23]. This is confirmed by the results of studies aimed at studying *L. murinus*, it was shown that representatives of this bacterial species are able to prevent the further development of intestinal ischemia caused by intestinal damage [24]. It was also demonstrated that two-week oral administration of this bacterial species to rats with spontaneous hypertension promoted the restoration of damaged vascular endothelial functions, as well as a decrease in blood pressure in rats, in addition, a decrease in the level of LPS in plasma was noted [25]. Thus, it can be said that representatives of the bacterial species *L. murinus* in the body are able to act as markers of a healthy state of the body. The results of our study showed an increase in the number of *L. murinus* in the intestines of a group of mice that were injected with LPS, relative to the control group.

The *Alistipes* genus of bacteria is known to be relatively new and not fully studied. *Alistipes* species are capable of producing short-chain fatty acids with anti-inflammatory effects. However, the abundance of *Alistipes* spp. is associated with aging and a number of gastrointestinal diseases, including IBD, liver cirrhosis, chronic fatigue syndrome, depression, anxiety, and autism [26]. However, it has been noted that this genus may protect against certain diseases [27].

In our study, we observed a higher abundance of *Alistipes communis*, *Alistipes senegalensis*, *Alistipes indistinctus*, *Alistipes dispar*, *Alistipes megaguti*, and *Alistipes finegoldii* in the LPS group compared to the control group. *Alistipes senegalensis JC50* has previously been shown to be involved in the development of IBD and metabolic disorders in humans. However, in a mouse model of colitis, it was shown that the abundance of this bacterial species can reduce inflammatory processes [28].

A study examining the effect of the bacterial species *Alistipes indistinctus* on the progression of non-alcoholic fatty liver disease demonstrated improved intestinal barrier function and inhibition of the LPS/TLR4/NF-κB pathway, which contributed to a reduction in liver inflammation [29]. This bacterium is also known to remove urates from the intestine, so it can be said that a high prevalence of *A. indistinctus* may be associated with a reduced risk of developing hyperuricemia [30]. Furthermore, mice receiving *A. indistinctus* orally in addition to a high-fat diet were completely protected from the development of insulin resistance and obesity [31]. *A. indistinctus* is a bacterial species that consumes a wide range of monosaccharides. In mice diagnosed with obesity, this bacterial species reduced blood sugar levels and carbohydrate availability [32]. Another study also found that a high-amylose diet in mice led to a decrease in *A. indistinctus* bacteria [33].

Previous studies have shown that mice transplanted with fecal microbiota from obese donors experienced increased levels of the potential pathobiont *Alistipes finegoldii*. A number of validation studies have shown that this bacterial species promoted the development of colon cancer [34]. Meanwhile, in a study examining the effect of DSS on colitis, this species reduced the severity of colitis [35]. A negative correlation was also observed between *A. finegoldii* and TNF-α levels after LPS injections [36]. In turn, for bacteria of the *A. finegoldii* species, an increase in the expression of intestinal IL-22 and Reg3γ in the colon was noted, under the influence of which the function of the intestinal barrier was restored [37]. Thus, literary data confirm that the bacterial species *Alistipes finegoldii* can be a pathobiont. Also, according to the results of our study, an increase in the abundance of the bacterial species *Bacteroides thetaiotaomicron*, *Bacteroides uniformis*, *Bacteroides nordii* was shown for the “LPS” group. It is known that *Bacteroides thetaiotaomicron* is a widespread commensal bacterium, the main metabolic products of which are: acetate, propionate and succinate [38]. Previous observations showed a positive correlation between the abundance of *B. theta* representatives and the alleviation of metabolic syndrome in the early and final stages of non-alcoholic fatty liver disease [39]. In addition, it was shown that representatives of the *B. theta* species are able to stop the development of TLR9-mediated colitis; in addition, they utilize *C. glabrata* from the intestine due to chitinase-like and mannosidase-like activity [40]. In turn, it has been shown that intestinal colonization with *B. theta* promotes thrombosis, exacerbates platelet hyperreactivity, and also leads to an increase in fecal L-tryptophan levels [41].

*Bacteroides uniformis* is a bacterial species with a high prevalence in the intestinal tract of healthy humans and mice [42]. *Bacteroides uniformis* has been shown to exert regulatory functions on intestinal homeostasis in animals; however, the mechanism by which it reduces the severity of colitis in mice remains unknown [43]. It has also been shown that increased abundance of *B. uniformis* and intragastric administration of cholic acid and chenodeoxycholic acid improved glucose and lipid metabolism in mice with type 2 diabetes mellitus by suppressing gluconeogenesis and lipolysis in the liver [44]. Results have shown that this bacterial species can improve exercise performance through gluconeogenesis [45].

The bacterial species *Bacteroides nordii* is a poorly studied representative of the pathogenic *B. fragilis* group, which consists of several strains with multiple drug resistance [46]. *B. nordii* is capable of influencing the metabolism of amino acids, as well as short-chain fatty acids [47].

*Duncaniella dubosii* bacteria are producers of short-chain fatty acids in the body [48]. The bacterial species *Duncaniella dubosii* plays an important role in linking tryptophan supplements with improving gut microbial diversity and balance. In addition, it is known that these bacteria are positively associated with immune modulation and promotes increased survival during brain tumor progression in a mouse model [49]. In addition, this bacterial species is likely associated with the development of autoimmune diseases [50].

Also in our study, an increase in the abundance of bacterial species was obtained: *Alkaliflexus* sp. *Ai-910*, *Wansuia hejianensis*, in the “LPS” group relative to the control.

*Akkermansia muciniphila* is associated with metabolic pathways and their regulation through the production of acetate and propionate, which have anti-inflammatory properties. These metabolites stimulate mucin synthesis, strengthen the intestinal barrier, and improve insulin sensitivity [51]. *A. muciniphila* reduces fat and triglyceride absorption, improves thermogenesis in adipose tissue, and reduces liver steatosis. These effects are associated with modulation of gene expression involved in lipogenesis and β-oxidation [52]. Modulation of physiological processes at the intestinal level is based on the ability of *A. muciniphila* to strengthen tight junctions between epithelial cells by increasing the expression of occludin and claudin proteins, which prevents the translocation of lipopolysaccharides (LPS) and toxins into the bloodstream, reducing systemic inflammation [53]. The formation of immune modulation is associated with the fact that the bacterium interacts with Toll-like receptors (TLR2/TLR4) and stimulates the production of anti-inflammatory cytokines (IL-10), suppressing the activity of proinflammatory ones (TNF-α, IL-6, NF-κB). This is important in autoimmune diseases such as multiple sclerosis and IBD [54,55]. Thus, the increase in *Akkermansia muciniphila* content with prebiotic supplement 1 may indicate a strengthening of the mucus barrier, a decrease in growth, and maintenance of lipid metabolism.

According to the results of the study, an increase in the number of *A. muciniphila* and *Anaerostipes rhamnosivorans* was observed in the intestines of mice receiving prebiotic supplement 1, relative to the LPS group. *Anaerostipes rhamnosivorans* is a gram-variable, strictly anaerobic bacterium that produces butyrate [56]. As is known, butyrate is a short-chain fatty acid and has anti-inflammatory properties [57]. The predominance of *Anaerostipes rhamnosivorans* in the context of taking prebiotic preparations ensures long-term nutrition of the intestine, as well as providing anti-inflammatory properties.

*Butyricimonas virosa* has been associated with intestinal diseases including diverticulitis and terminal ileitis in humans [58]. The sensitivity of the bacterium to bile acids and oxygen exposure during intestinal barrier dysfunction may further explain its altered abundance in human IBD [59]. Competition with other microbial taxa also influences the abundance of this species, *B. virosa* thriving in environments where it can metabolize dietary fiber to butyrate, a process inhibited by dysbiosis [60]. In our study, prebiotic supplement 1 increased *B. virosa* abundance compared to the LPS group, consistent with its reported butyrate-producing capacity.

The bacterial species *Muribaculum intestinale* is involved in carbohydrate metabolism by fermenting complex polysaccharides (e.g., dietary fiber and mucins) to short-chain fatty acids (SCFAs) such as acetate and butyrate, which are critical for maintaining intestinal barrier homeostasis and immune regulation [61]. A link between antibiotic exposure and changes in the levels of this bacterial species in the mouse intestine has been previously shown [62]. The bacterium produces cardiolipin (MiCL-1), which activates TLR2/TLR1-dependent pathways and induces the production of proinflammatory cytokines (TNF-α, IL-6, IL-23). This indicates a role in shaping the immune response, although excess may promote inflammation [63]. In murine inflammatory bowel diseases such as Crohn’s disease, a decrease in *M. intestinale* is observed in parallel with an increase in proinflammatory taxa (e.g., *Bacteroides* and *Prevotella*) [64].

In this study, along with the increase in this bacterial species in the LPS group, compared to prebiotic supplement 1, the abundance of the species *Duncaniella dubosii*, *Ligilactobacillus murinus*, *Alkaliflexus* sp. *Ai-910*, *Phocaeicola vulgatu*, *Parabacteroides distasonis* and *Coprococcus* sp. *ART55/1* also increased.

*Phocaeicola vulgatus* is a pathogenic anaerobic bacterium in the colon that can cause infections in humans [65], involved in the fermentation of polysaccharides, producing SCFA such as acetate and propionate [66]. *P. vulgatus* has also been associated with protection against colitis and metabolic disorders; however, overgrowth can promote inflammatory bowel disease in genetically susceptible hosts [67].

*Parabacteroides distasonis* is a commensal with anti-inflammatory and metabolic regulatory functions of Gram-negative anaerobic bacteria that commonly colonize the gastrointestinal tract of many species. There is no clear opinion regarding the impact of *Parabacteroides* on human health; members of this genus can have both positive and negative effects [68].

The genus *Blautia* bacteria plays an important role in metabolism, including the fermentation of carbohydrates to produce short-chain fatty acids such as acetate, which affects host health [69]. In our study, an increase in *Blautia sp*. *NBRC 113351* was observed in the prebiotic supplement 1 group relative to the LPS group.

Decreased levels of *Coprococcus* bacteria are associated with depression, anxiety disorders, and Parkinson’s disease. This is explained by a decrease in the production of butyrate and the dopamine metabolite DOPAC, which affects the functioning of the central nervous system [70]. According to a study [71], an increased number of *Coprococcus* sp. *ART55/1* was associated with an increased risk of developing primary membranous nephropathy, indicating a possible link between this strain and the development of the disease, but the exact mechanism is unclear.

The bacterial species *Longicatena caecimuris* belongs to the phylum Firmicutes, which play a key role in carbohydrate fermentation and SCFA production [72]. In a study [73], *L. caecimuris* was associated with a positive response to immune checkpoint inhibitor (ICI) therapy in patients with metastatic melanoma. In Crohn’s disease and ulcerative colitis, a decrease in the diversity of potentially beneficial species such as *L. caecimuris* is observed [74]. In mice, *L. caecimuris* is associated with the regulation of inflammatory processes, although the exact mechanisms require further study [72], while in humans its presence may affect immune homeostasis given the association of other representatives of the Erysipelotrichaceae with autoimmune diseases [75]. The level of this bacterial species in our study was reduced in the group receiving prebiotic supplement 1 relative to the LPS group.

Our study also showed that the number of *L. murinus* bacteria was significantly reduced in the group where mice received prebiotic supplement 2, which may indicate that these prebiotics are not able to stimulate the growth of *L. murinus* specifically.

*Butyricimonas* is a genus of Gram-negative anaerobic bacteria of the Odoribacteraceae family that are present in the intestinal tract of mammals, including mice, rats, and humans [76]. There is evidence that *Butyricimonas* species participate in commensal homeostasis between the gut microbiota and the host and have beneficial effects on host energy metabolism [77]. For example, Heetae Lee et al. showed that *B. virosa* supplementation in obese mice normalized body weight, glucose levels, and other parameters [78]. Our results show that prebiotic supplementation increased the relative abundance of *B. virosa*, consistent with shifts in microbiome composition observed after prebiotic supplementation.

*Vescimonas coprocola* is a newly identified species belonging to the genus *Vescimonas*, which was first described in a 2021 study [79]. The role of these bacteria in microbiome function is not yet determined; however, our study showed that prebiotic supplementation increased the abundance of these bacteria compared to the LPS group, which may indicate a potential beneficial effect of *V. coprocola*.

The anaerobic, Gram-positive, rod-shaped bacterium *Flavonifractor plautii* (formerly *Eubacterium plautii*) belongs to the *Clostridiales* family. This species of bacteria is a common member of the gut microbiome and is also known to be involved in catechin metabolism [80]. A study by Ayane Mikami showed that 10-day oral administration of *F. plautii* to mice with colitis resulted in reduced intestinal inflammation [81], which is consistent with our findings.

*Alkaliflexus* sp. *Ai-910* bacteria are uncultivated and very little is known about their role in the gut microbiome [82]. Our study showed that the number of *Alkaliflexus*. sp. *Ai-910* decreased in the Prebiotic supplement 2 group, which warrants further investigation into its role in the gut microbiome. There is a possible negative impact of these bacteria on the gut microbiome, although this hypothesis requires additional separate studies. As in the group of mice fed prebiotic 1, an increase in the number of *A. rhamnosivorans* bacteria was shown in the group of mice fed prebiotic 2 compared to the LPS group, indicating a beneficial effect of the prebiotics used on the gut microbiome.

*Dysosmobacter welbionis* is a recently identified butyrate-producing commensal bacterium. A study by Tiphaine Le Roy et al. showed that administration of live *D. welbionis* to mice partially counteracted the development of obesity, increased fat mass, insulin resistance, white adipose tissue hypertrophy, and inflammation. In addition, administration of these bacteria protected mice from brown adipose tissue inflammation [83]. Our results also demonstrate an increase in the relative abundance of bacteria in the Prebiotic supplement 2 group compared to LPS, suggesting a beneficial effect of prebiotic supplementation on the composition of the gut microbiome. The increase in the relative abundance of *Dysosmobacter welbionis* in our study may be associated with improved glucose metabolism, insulin sensitivity and intestinal integrity.

This study has a methodological limitation. There is no control groups receiving ‘supplement 1 without LPS’ and ‘supplement 2 without LPS’. This prevents us from differentiating the direct anti-inflammatory effect from the indirect effect associated with general microbiome modulation.

## 5. Conclusions

*Fucus vesiculosus* and *Laminaria digitata*-derived prebiotics significantly altered gut microbiome composition and predicted metabolic pathways in LPS-induced mice. These modulations included reduced glycolysis activity characteristic of dysbiosis correction. The findings support the therapeutic potential of algae-based prebiotics for inflammatory gut dysbiosis.

## Figures and Tables

**Table 1:** Chemical Composition of Prebiotic Preparations.

Composition		Prebiotic Supplement 1	Prebiotic Supplement 2
Non-cellulosic polysaccharides, mg/g dry sample	Xylose	1.08	10.81
	Mannose	2.11	21.12
	Galactose	1.76	17.56
	Glucose	8.68	86.79
	Fucose	8.15	81.55
	GlcA	1.99	19.86
	GalA	1.43	14.3
Elemental composition, mg/100 g	Ag	0	0
	Al	1.0545	10.54
	As	0.091	0.91
	B	0.0781	0.78
	Ba	0.3093	3.09
	Be	0	0
	Ca	135.27	1352.7
	Cd	0	0.38
	Co	0	0
	Cr	0.0264	0.26
	Cu	0	0
	Fe	2.9796	29.8
	K	145.145	1451.45
	Mg	68.4305	684.31
	Mn	1.1385	11.38
	Mo	0	0
	Na	103.1915	1031.92
	Ni	0	0
	P	5.9462	59.46
	Pb	0	0
	S	190.1	1901
	Sb	0	0
	Se	0	0
	Si	3.7289	37.29
	Ti	0.0911	0.91
	Tl	0	0
	V	0	0
	Zn	0.1852	1.85

**Table 2:** Histological examination of the intestine.

Pile Height, µm	Villi Width, µm	Crypt Depth, µm
Control	286.6 ± 16.1	34.5 ± 4.1
LPS	296.1 ± 21.7	22.2 ± 2.3
Prebiotic supplement 1	278.8 ± 17.7	32.2 ± 3.3
Prebiotic supplement 2	370.9 ± 24.2	35.6 ± 3.1

**Table 3:** The number of bacteria and measures of their diversity.

Group	Observed Species	Shannon Index
Control	55	2.212
LPS	69	2.706
Prebiotic supplement 1	74	2.521
Prebiotic supplement 2	77	2.977

## Data Availability

Sequencing data are available in the NCBI BioProject database (BioProjectID: PRJNA1292815).

## Abbreviations

The following abbreviations are used in this manuscript:

LPS	Lipopolysaccharide
UDB	Union Database
cDNA	Complementary DNA
PCR	Polymerase chain reaction
dsDNA	Double-Stranded DNA
ssDNA	Single-stranded DNA
PE	Paired-End
FCL	Flow Cell Large
FCS	Flow Cell Small
MetaPhlAn	Metagenomic Phylogenetic Analysis
MaAsLin	Multivariate Analysis by Linear Models
